# Hedgehog signaling promotes T_H_2 differentiation in naive human CD4 T cells

**DOI:** 10.1016/j.jaci.2019.07.011

**Published:** 2019-11

**Authors:** Diana C. Yánez, Ching-In Lau, Mira Manilal Chawda, Susan Ross, Anna L. Furmanski, Tessa Crompton

**Affiliations:** a UCL Great Ormond Street Institute of Child Health, London, United Kingdom; b School of Medicine, Universidad San Francisco de Quito, Quito, Ecuador; c School of Life Sciences, University of Bedfordshire, Luton, United Kingdom

To the Editor:

Hedgehog (Hh) proteins are intercellular signaling molecules that control development and tissue homeostasis. They also regulate thymocyte development and peripheral T-cell activation in mice and human subjects and have recently been shown to promote T_H_2 differentiation and function in mice.^1^, ^2^, ^3^, ^4^ Sonic Hedgehog homologue (SHH) is involved in homeostasis of many epithelial tissues, and because these tissues are the sites of allergic disease, it is important to understand how Hh signaling influences human CD4 T_H_ differentiation. Here we show that Hh signaling promotes human T_H_2 differentiation by using materials and methods described in Furmanski et al^3^ and Yanez et al^5^ and in the Methods section in this article's Online Repository at www.jacionline.org.

We used quantitative RT-PCR to evaluate gene expression of components of the Hh signaling pathway in naive human CD4 T cells stimulated for 48 hours in T_H_0-, T_H_1-, or T_H_2-polarizing conditions. Expression levels of the Hh-responsive transcription factors glioma-associated oncogene 1 *(GLI1)* and *GLI2* and the Hh cell-surface receptor patched 1 *(PTCH1)* were greater in CD4 T cells cultured under T_H_2-skewing conditions compared with those cultured under T_H_0 or T_H_1 conditions (Fig 1, *A*), suggesting that Hh signaling is involved in human T_H_ differentiation or function. Because *GLI1* and *PTCH1* are Hh target genes, their greater expression in T_H_2-differentiated cells indicates that this population has overall greater Hh-mediated transcription.

**Fig 1:**
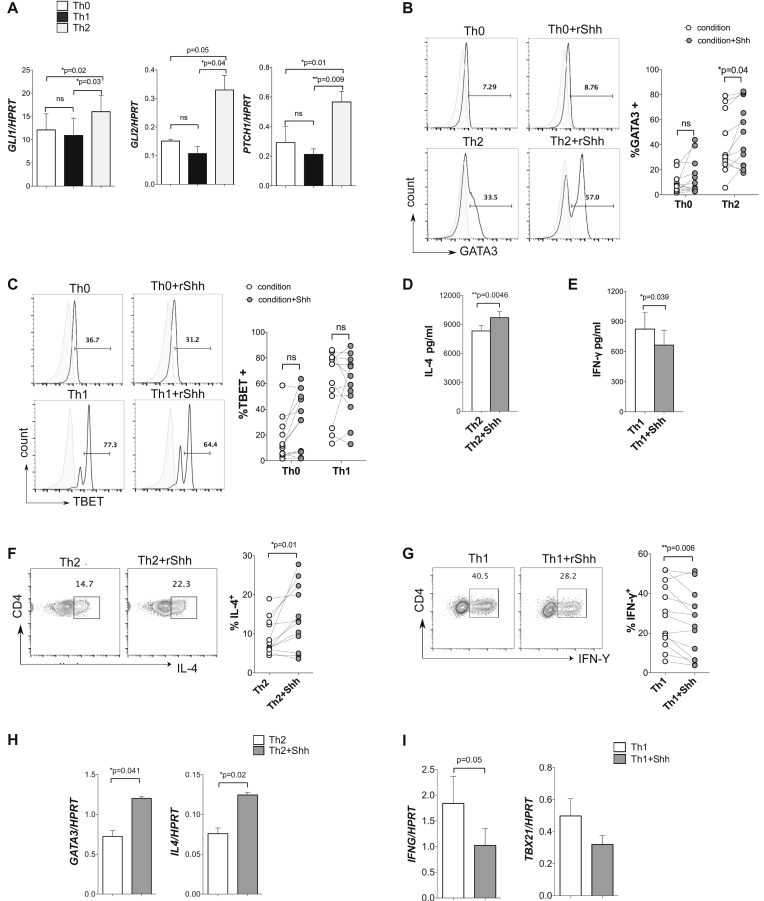
Shh treatment increases T_H_2 differentiation *in vitro*. Naive CD4 T cells (n = 12 donors) stimulated under T_H_-skewing conditions with or without rShh **(B-I)** analyzed at 48 hours **(A)**, at day 4 **(B-E)**, and at day 7 plus restimulation **(F-I)** are shown. Plots indicate means ± SEMs; each *point* represents an individual donor. Fig 1, *A*, Gene expression (quantitative RT-PCR; n = 3). FACS histograms show intracellular expression (gated on CD4^+^ cells) of GATA-3 (Fig 1, *B*) and T-bet (Fig 1, *C*). *Gray overlays* show control stain. Scatterplots show percentages of positive cells. Fig 1, *D* and *E*, Cytokine concentration (ELISA) in supernatants. Fig 1, *F* and *G*, FACS plots show CD4 and intracellular cytokine expression. Scatterplots show cytokine-positive percentages. Fig 1, *H* and *I*, Gene expression (quantitative RT-PCR; n = 3). *P < .05 and **P < .01, paired 2-tailed *t* test. *ns*, Not significant.

To test the influence of SHH signaling on T_H_ differentiation, we stimulated purified naive human CD4 T cells from 12 independent, randomly selected anonymous donors for 4 days under skewing conditions with or without a single dose of recombinant Shh (rShh). Treatment with rShh significantly enhanced expression of the T_H_2 transcription factor GATA-3 in cells stimulated under T_H_2 conditions, whereas GATA-3 expression under T_H_0 conditions and T-bet expression under T_H_0 or T_H_1 conditions were not affected (Fig 1, *B* and *C*). Treatment of T_H_2-skewing cultures with rShh also increased the concentration of IL-4 in supernatants after 4 days of culture compared with control T_H_2-skewing cultures (Fig 1, *D*). Interestingly, the concentration of IFN-γ was lower when rShh was added compared with control T_H_1 cultures (Fig 1, *E*). After 7 days of culture and anti-CD3/CD28 restimulation, the proportion of CD4 T cells that expressed IL-4 was significantly increased in the presence of rShh under T_H_2 conditions (Fig 1, *F*). In contrast, the percentage of cells that expressed IFN-γ was reduced in T_H_1 plus rShh cultures compared with T_H_1 cells (Fig 1, *G*). Shh treatment increased *GATA3* and *IL4* expression in T_H_2 cultures (Fig 1, *H*), whereas rShh treatment decreased *IFNG* and *TBX21* (T-bet) expression in T_H_1 cultures (Fig 1, *I*). These data indicate that Hh signaling promotes T_H_2 differentiation in human CD4 T cells, with simultaneous repression of IFN-γ and T-bet.

We then investigated whether pharmacologic inhibition of the Hh signaling pathway by treatment with an inhibitor of the nonredundant Hh signal transduction molecule smoothened (SMO; PF-04449913) would impair T_H_2 differentiation.^6^ The proportion of cells that expressed the T_H_1 lineage–specific transcription factor T-bet was not affected by SMO inhibitor treatment under skewing conditions (Fig 2, *A*). Likewise, no differences were found in expression of GATA-3 under neutral or T_H_1 conditions (Fig 2, *B*). However, SMO inhibitor treatment significantly reduced the proportion of CD4 T cells that expressed GATA-3 and Ki-67 (a marker of proliferation) when cultured under T_H_2-skewing conditions (Fig 2, *B* and *C*). SMO inhibition did not affect the percentage of cells that expressed IFN-γ under T_H_1 conditions, and as expected, IL-4 expression was low under T_H_1 conditions in both control and SMO inhibitor–treated cultures (Fig 2, *D*). However, when cultured under T_H_2 conditions, the percentage of cells that expressed IL-4 was significantly reduced by SMO inhibitor treatment (Fig 2, *E*). Analysis of cytokine concentrations in culture supernatants by means of ELISA showed that IFN-γ levels were similar in both groups under T_H_1 conditions (Fig 2, *F*), but under T_H_2 conditions, significantly lower concentrations of IL-4 were found in the SMO inhibitor group compared with the control group (Fig 2, *G*). Finally, we investigated transcript levels of *IL4* and *IFNG* by using quantitative RT-PCR. In T_H_2-skewed cells *IL4* expression was significantly lower in SMO inhibitor–treated cultures than control cultures (Fig 2, *H*), whereas *IFNG* transcript levels were not different between groups under T_H_1 conditions (Fig 2, *I*). Taken together, these analyses indicate that attenuation of Hh signal transduction by treatment with the SMO inhibitor reduced T_H_2 differentiation but did not affect T_H_1 fate.

**Fig 2:**
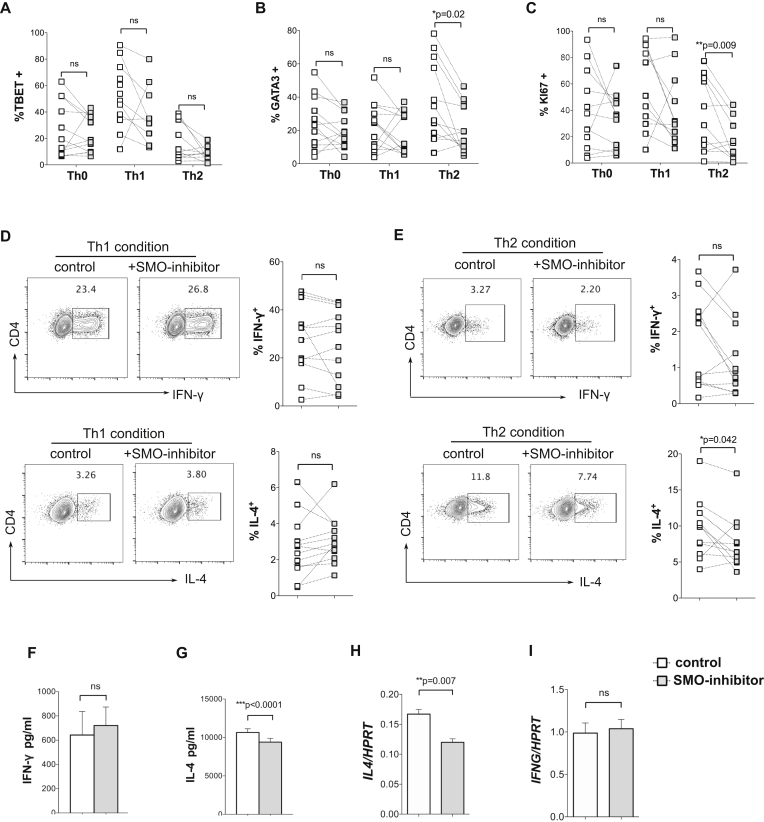
SMO inhibition decreases T_H_2 differentiation *in vitro*. Naive CD4 T cells (n = 12 donors) stimulated under T_H_-skewing conditions with SMO inhibitor *(gray squares)* or DMSO (control; *open bars/squares*) on day 4 (**A-C, F,** and **G**) and day 7 plus restimulation (**D, E, H,** and **I**). Scatterplots show means ± SEMs; each *point* represents an individual donor. Fig 2, *A-C*, Percentage of CD4^+^ cells that were positive for intracellular staining against T-bet (Fig 2, *A*), GATA-3 (Fig 2, *B*), and Ki-67 (Fig 2, *C*). Fig 2, *D* and *E*, FACS plots show expression of CD4 and intracellular IFN-γ *(upper plots)* or intracellular IL-4 *(lower plots)* in cells cultured under T_H_1 (Fig 2, *D*) or T_H_2 (Fig 2, *E*) conditions. Scatterplots show percentages of CD4^+^ cells that stained positive with the stated cytokine. Fig 2, *F* and *G*, Cytokine concentration (ELISA) in supernatants from T_H_1 (Fig 2, *F*) and T_H_2 (Fig 2, *G*) cultures. Fig 2, *H* and *I*, Gene expression (quantitative RT-PCR) in cells from T_H_2 (Fig 2, *H*) and T_H_1 (Fig 2, *I*) cultures (3 random donors). *P < .05, **P < .01, and ***P < .001, paired 2-tailed *t* test. *ns*, Not significant.

Here we show that Hh signaling promotes T_H_2 differentiation in human CD4 T cells. We found that treatment of naive CD4 T cells with rShh under T_H_2-skewing conditions increased expression of the transcription factor GATA-3, a reliable indicator of T_H_2 transcriptional identity. In support of this, *IL4* expression was enhanced and IL-4 cytokine production was increased in T_H_2 cultures on treatment with rShh. In contrast, rShh treatment antagonized T_H_1 differentiation in T_H_1 cultures, leading to lower *IFNG* and *TBX21* expression and a lower proportion of cells expressing intracellular IFN-γ. Attenuation of Hh signal transduction by pharmacologic SMO inhibition reduced T_H_2 differentiation: both *GATA3* expression and *IL4* expression were significantly decreased.

In murine T_H_ differentiation Hh signaling promotes T_H_2 differentiation, skewing the overall pattern of transcription to a T_H_2-like profile, and *Il4* is a GLI2 target gene in murine T cells.^3^ Importantly, Hh pathway activation in T cells has physiologic relevance in a murine model of allergic asthma because by favoring T_H_2 polarization and cytokine production, it contributes to disease severity.^3^, ^7^

In human subjects a genome-wide association study linked components of the Hh signaling pathway to allergic asthma,^8^ and a recent study found that children with asthma presented with greater levels of SHH in airway epithelia than healthy control subjects.^9^

Here we provide *in vitro* evidence that Hh signaling enhances T_H_2 differentiation in human CD4 T cells. One strength of our study is that our experiments were performed with cells isolated from 12 different unknown leukocyte cone donors, and we obtained consistent experimental results from all donors independent of their age or sex (of which we had no knowledge). A weakness of our study is that it was limited to *in vitro* experimentation. In the future, it will be interesting to assess the T_H_ differentiation status of T-cell populations isolated from samples from patients with asthma to obtain further *ex vivo* evidence that Hh signaling is involved in human T_H_2 responses. This will be important to our understanding of human atopic diseases, such as asthma, in which T_H_2 T-cell responses drive disease.

## Methods

### Human naive CD4 purification and culture

Human PBMCs were freshly isolated from randomly selected, unknown leukocyte cone donors (UK National Health Service [NHS] Blood and Transplant Centre) by means of gradient centrifugation with Lymphoprep (Axis Shield, Oslo, Norway). Donors to the UK NHS Blood and Transplant Centre are aged between 17 and 65 years, and we had no knowledge of their age, sex, or identity. Ethical approval was authorized by the local NHS Research Ethics Committee.

Naive CD4 T cells (CD3^+^CD4^+^CD45RA^+^CD45RO^−^) were magnetic bead purified from PBMCs by using the EasySep Isolation Kit (STEMCELL Technologies, Vancouver, British Columbia, Canada). The purity of naive CD4 T cells was analyzed by using flow cytometry and exceeded 95%. After magnetic bead isolation, naive CD4 T cells were rested for 3 to 5 hours and then plated in 96-well round plates at 1 × 10^6^ cells/mL. Cells were stimulated in complete RPMI (supplemented with 10% FBS, 1% penicillin-streptomycin, and 10^−5^ mol/L 2-mercaptoethanol) with 5 μg/mL plate-bound anti-CD3 antibody (clone UCHT1) and anti-CD28 antibody (eBioscience, San Diego, Calif). For T_H_0 conditions, no cytokines were added. For T_H_1 conditions, anti–IL-4 (5 μg/mL), rIL-12 (20 ng/mL), and rIFN-γ (10 ng/mL) were added. For T_H_2 conditions, anti–IFN-γ (2.5 μg/mL) and rIL-4 (20 ng/mL) were added.

After 4 days, cells were expanded in human rIL-2 (100 U/mL) for 3 days in fresh medium containing the same skewing cytokines and neutralizing antibodies but in the absence of anti-CD3 and CD28 stimulation. Cells were then restimulated for 16 hours by addition of soluble anti-CD3 and anti-CD28 (1 μg/mL) before gene expression and cytokine analysis. Where stated, rShh (R&D Systems, Minneapolis, Minn) was added at a final concentration of 0.5 μg/mL at the initiation of culture and again on day 4, when the medium was changed and the cells were expanded by addition of rIL-2.

Where stated, SMO inhibitor (PF-04449913 [Pfizer, New York, NY] dissolved in dimethyl sulfoxide [DMSO]) was added to cultures for a final concentration of 0.374 μg/mL, and an equivalent concentration of DMSO alone was added to the control wells (DMSO at 1:10,000 final dilution). This treatment or control was added to the corresponding wells every day until the end of the experiment.

For intracellular cytokine staining, CD4 T cells were stimulated for 4 hours with 50 ng/mL phorbol 12-myristate 13-acetate (Sigma-Aldrich, St Louis, Mo), 500 ng/mL ionomycin (Sigma-Aldrich), and 3 μg/mL Brefeldin A (eBioscience).

### Flow cytometry

Cells were stained with combinations of directly conjugated antibodies from Thermo Fisher (Waltham, Mass) or BioLegend (San Diego, Calif) in fluorescence-activated cell sorting (FACS) buffer (5% FBS and 0.01% sodium azide in 1× PBS) acquired on a C6 Accuri flow cytometer (BD Biosciences, San Jose, Calif) and analyzed with FlowJo software (version 10.6; TreeStar, Ashland, Ore). For intracellular staining, CD4 T cells were stained with anti-CD4 for cell-surface staining and then incubated with Fixation/Permeabilization solution (eBioscience) for 20 minutes in the dark. After this, cells were washed twice with permeabilization buffer and then stained with specific antibodies in permeabilization buffer for 40 minutes. After incubation, cells were washed with permeabilization buffer and resuspended in FACS buffer for FACS analysis.

### ELISA

IFN-γ and IL-4 cytokines were measured with Ready-Set-Go! Kits (eBioscience), according to the manufacturer's instructions.

### Quantitative RT-PCR

RNA was extracted with the PicoPure Kit (Applied Biosystems, Foster City, Calif). cDNA was synthesized by using the High Capacity cDNA Reverse Transcription Kit (Applied Biosystems) and were analyzed on an iCycler (Bio-Rad Laboratories, Hercules, Calif) with SYBR Green Supermix (Bio-Rad Laboratories), according to the manufacturer's guidelines. RNA levels obtained from each sample were measured relative to the housekeeping gene hypoxanthine phosphoribosyltransferase *(HPRT)*. All primers were purchased from Qiagen (Hilden, Germany).

### Statistical analysis

The paired 2-tailed Student *t* test was used for statistical analysis for comparison of *in vitro* treatment of cells from a given subject.
